# Emergency Obstetric Hysterectomy, the Histopathological Perspective: A Cross-Sectional Study From a Tertiary Care Hospital

**DOI:** 10.7759/cureus.9094

**Published:** 2020-07-09

**Authors:** Ruqaiya Shahid, Hina Abbas, Shazia Mumtaz, Muhammad Furqan Bari, Naseem Ahmed, Shaima Memon, Tazeen Raja, Kartar Dawani

**Affiliations:** 1 Pathology, Dow International Medical College, Dow University of Health Sciences, Karachi, PAK; 2 Hematology, Dow Medical College, Dow University of Health Sciences, Karachi, PAK; 3 Pathology, Dow University of Health Sciences, Karachi, PAK; 4 Pathology, Dow Medical College, Dow University of Health Sciences, Karachi, PAK

**Keywords:** hysterectomy, obstetrics, placenta accreta, placenta previa, abruptio placentae

## Abstract

Introduction

Emergency obstetric hysterectomy (EOH) is a life-saving procedure which involves the surgical removal of uterus and is usually performed for uncontrollable maternal hemorrhage when all other conservative management has failed. This study was conducted to evaluate the histopathological findings in the EOH specimen received in the department of pathology.

Methods

This hospital-based cross-sectional study was conducted in the Histopathology Laboratory, Department of Pathology, and Dow Medical College (DMC) from September 2017 to December 2018. The histopathological findings in the EOH specimen were recorded and data was analyzed.

Results

Ninety-six cases of EOH were received. The incidence of emergency obstetric hysterectomy was 58.37/10,000 deliveries. The mean age of patients was 30.59 years (range 20-45 years). The main histopathological findings were placenta accreta spectrum in 61 (63.54%) cases, cervical tear in eight (8.33%), uterine rupture in seven (7.29%) and endomyometritis in six (6.25%) cases. In the placenta accreta spectrum, placenta accreta was the most frequent diagnosis in 23 (23.96%) of cases, placenta increta in 17 (17.71%), placenta percreta in 10 (10.42%) cases. Seven (7.29%) cases of placenta percreta and four (4.17%) cases of placenta accreta were diagnosed in association with placenta previa. Twenty placentas were received with the hysterectomies, of these eight (40%) placentas showed infarction and six (30%) had intervillous fibrin, both findings were suggestive of uteroplacental insufficiency, while three (15%) placentas had normal histology. Ovaries were received with the hysterectomies in 11 (11.46%) cases. Mature cystic teratoma was diagnosed in two (2.08%) ovaries while the majority of ovaries were normal on histology.

Conclusion

Placenta accreta spectrum is the leading histopathological finding in the EOH specimen. Regular antenatal follow-up and radiological examination of pregnant women is inferred to prevent obstetric complications and near-miss event of EOH. Further research is recommended to confirm the findings in placenta. Ovarian conservation is suggested in patients undergoing EOH with no clinical and surgical indication for oophorectomy.

## Introduction

Emergency obstetric hysterectomy (EOH) is defined as the surgical removal of uterus either at the time of vaginal or caesarean delivery or within puerperium period and is usually performed due to excessive obstetric hemorrhage. It is a lifesaving procedure carried out, when all other measures and interventions to secure maternal life have failed and maternal life loss becomes inevitable [1].

Obstetric causes account for a high maternal mortality and according to the World Health Organization (WHO) obstetric causes are responsible for 73% of all maternal deaths; whereas intra-partum and post-partum hemorrhage is the leading direct cause of maternal mortality worldwide [2]. Sustainable Development Goal 3.1, by WHO, aims to reduce the maternal mortality ratio (MMR) to less than 70/100,000 live births by 2030 [3]. Pakistan belongs to the South Asian region that had an MMR of 178/100,000 deliveries in 2019 [4,5]. Most of the causes of maternal death are preventable; so as to decrease the MMR it is imperative to manage the obstetric cases effectively [5].

EOH is classified as “maternal near miss” event by WHO; the mother barely survives the pregnancy and its complications, but loses her uterus [6]. Although, EOH is a life-saving procedure, it carries a high risk of maternal and fetal mortality and procedural complications. Maternal mortality rate reported following EOH is 9% in a study from Pakistan, 17.7% from India and 14.3% from Nigeria, while the perinatal mortality rate was 53.7% from Pakistan, 37.5% from India and 64.3% from Nigeria. Some of the post-operative complications of EOH reported in literature are wound sepsis, acute lung injury, urinary tract injury, vesico-vaginal fistula and disseminated intravascular coagulation [1,7,8]. EOH results in an end to maternal fertility, this may have severe distressing effects on the woman ranging from physical pain to post traumatic stress disorder and psychological depression [9].

There are many causes of intra-partum and post-partum hemorrhage that lead to EOH. These include placenta accreta and its spectrum, placental abruption, trauma to the uterus and cervix at the time of delivery, uterus rupture and uterine inertia and placental site subinvolution [10].

Histopathological examination of the EOH specimens is necessary as it determines the true cause of obstetric hysterectomy and also confirms the clinical and radiological indications. Evaluation of the histopathological findings in the EOH specimens will help in addressing the root causes of EOH, and will provide future direction for improvement in the standard obstetric care, to prevent maternal morbidity and mortality and preclude near miss event of EOH.

The objective of the study was to evaluate the histopathological findings in the specimen of emergency obstetric hysterectomies received in the Department of Pathology from September 2017 to December 2018.

## Materials and methods

This retrospective, cross-sectional study was conducted at the Department of Pathology, Dow Medical College from September 2017 to December 2018. This was a single-center study. The Histopathology Laboratory of the department is affiliated with and receives specimen from Civil Hospital, Karachi which is a tertiary care government hospital. All the specimens that are received in the laboratory are directed for routine gross examination, where a pathologist takes representative sections and submits them for processing. The glass slides are prepared, examined by the pathologist and a diagnosis is rendered. In case of any uncertainty, opinion is sought from fellow pathologists. For this study the data of all the hysterectomies received in the department during the study period were retrieved. Histopathology reports were retrieved from the computer data system and reviewed and relevant information was entered by the first and the second authors. Data of patients undergoing EOH were included and data of patients with hysterectomies for gynecological causes were excluded. In case of incomplete data or query, the glass slides were reviewed and the gross specimens of EOH were re-examined by the first author. The variables recorded were: age of the patient, clinical information provided by the surgeon, histopathological findings in the uterus, fallopian tubes, ovaries and placentas. Microsoft Excel and IBM SPSS Statistics, version 20 (IBM Corp., Armonk, NY, USA) softwares were used for data entry and analysis. Mean was taken for continuous variable, i.e., age. Frequency and percentages were calculated for the categorical variables, i.e., the histopathological findings in the uterus, ovaries, fallopian tubes and placentas.

The definitions of the obstetric conditions which were followed for the diagnosis and reporting are: Placenta accreta: it is an abnormal adherence of placenta to the uterine wall, and is characterized microscopically by the lack of decidua between the placenta and the myometrium. Placenta increta is diagnosed when placenta is invading into the myometrium, and is determined by gross and microscopic examination [10]. In placenta percreta, placenta invades full thickness through the myometrium. Placenta previa is implantation of placenta into the lower uterine segment [10]. Uterus rupture is defined as complete rupture of myometrium, peritoneum and fetal membranes and is histologically characterized by full thickness disruption of the myometrium [11]. Uterine inertia or atony results due to failure of the uterine smooth muscles to contract; whereas, placental site subinvolution is identified by large ectatic and uninvoluted vessels at the placental site [10]. Trauma to the uterus in the form of cervical tears and rupture, at the time of delivery, was indicated on the provided clinical information form and was confirmed on gross and microscopic examination of the uterus.

Placentas including disc, umbilical cord and fetal membranes were examined for any gross abnormalities such as invasiveness into the uterine wall, hematomas, infarction, etc. Microscopically placentas were studied for: 1) Inflammatory conditions such as chorioamnionitis, funisitis, chorionic villitis and intervillositis. 2) Vascular lesions such as infarction. 3) Placental microscopic findings such as intervillous fibrin. 4) Anatomic abnormalities such as invasiveness of placenta into the myometrium and umbilical vessels [12].

Approval from the Institutional Review Board was taken before the commencement of the study.

## Results

Ninety-six EOH specimens were received and included in the study. The total number of deliveries at Civil Hospital, Karachi during the study period of 16 months was 16,446. The incidence of EOH was 58.37/10,000 deliveries. Mean age of the patients was 30.59 years (±4.62, range 20-45 years) and most of the patients were 26-35 years of age (Table 1).

**Table 1:** Age group distribution of patients with emergency obstetric hysterectomies.

Age group	Frequency	Percentage
15-25 years	13	13.5%
26-35 years	74	77.1%
36-45 years	9	9.4%
Total	96	100.0%

The results of the histopathological findings in EOH specimen are presented in Table 2.

**Table 2:** Histopathologic diagnosis in the emergency obstetric hysterectomies.

Histopathological diagnosis in uteruses (n = 96)	Frequency	Percentage
Placenta accreta	23	23.96%
Placenta Increta	17	17.71%
Placenta percreta	10	10.42%
Placenta percreta with previa	7	7.29%
Placenta accreta with previa	4	4.17%
Placenta previa	2	2.08%
Placenta previa with leiomyoma	1	1.04%
Abruptio Placentae	1	1.04%
Placental site sub-involution	1	1.04%
Cervical tear	8	8.33%
Uterus rupture during delivery	7	7.29%
Endomyometritis	6	6.25%
Uterine inertia	2	2.08%
Transverse fetal lie with cord prolapse	1	1.04%
Ectopic pregnancy in uterine horn	1	1.04%
No cause identified for post-partum hemorrhage	5	5.21%
Total	96	100%

**Figure 1:**
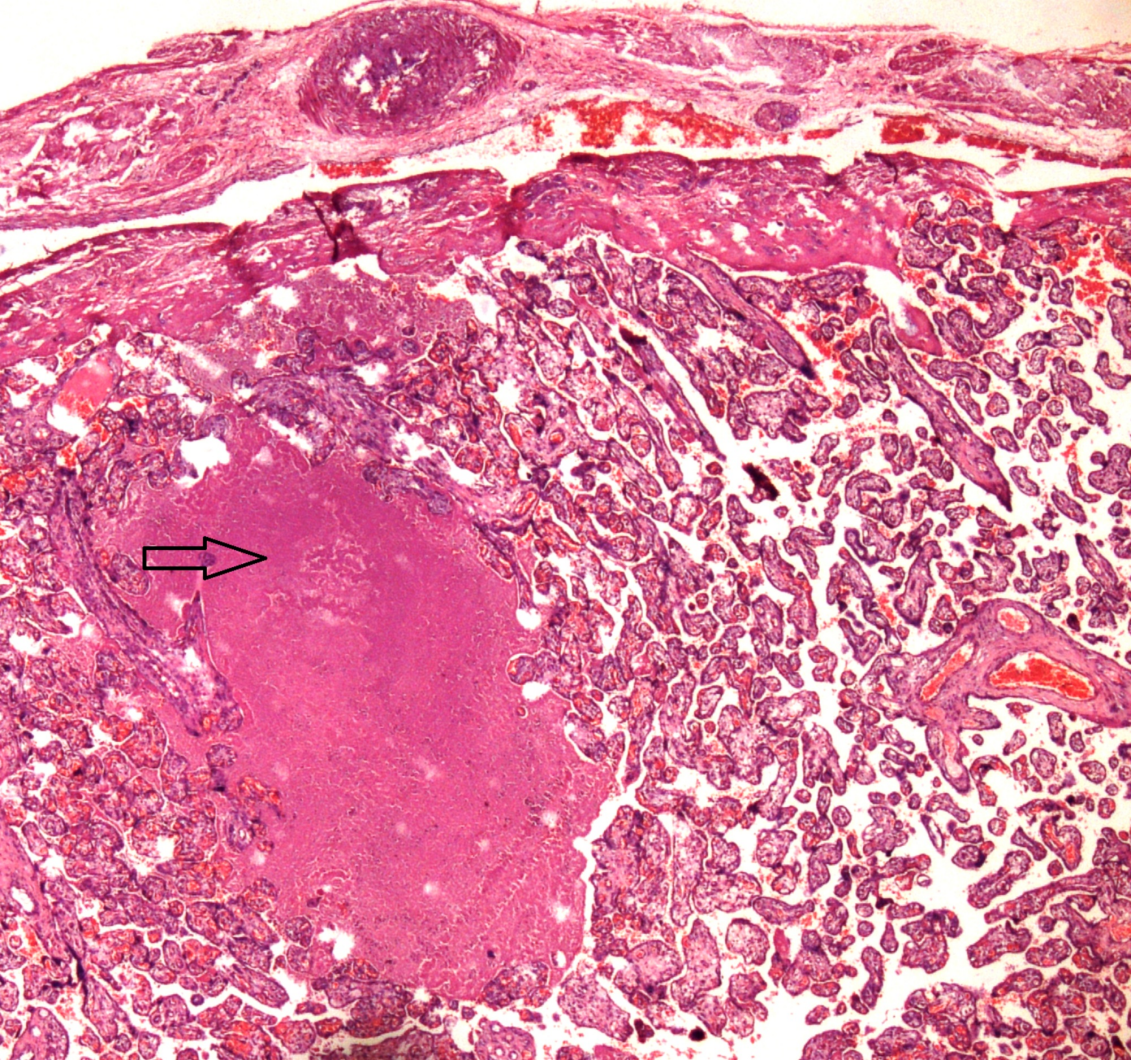
Placenta percreta. Placenta has invaded full thickness through the myometrium; a focus of intervillous fibrin is highlighted by the arrow.

**Figure 2:**
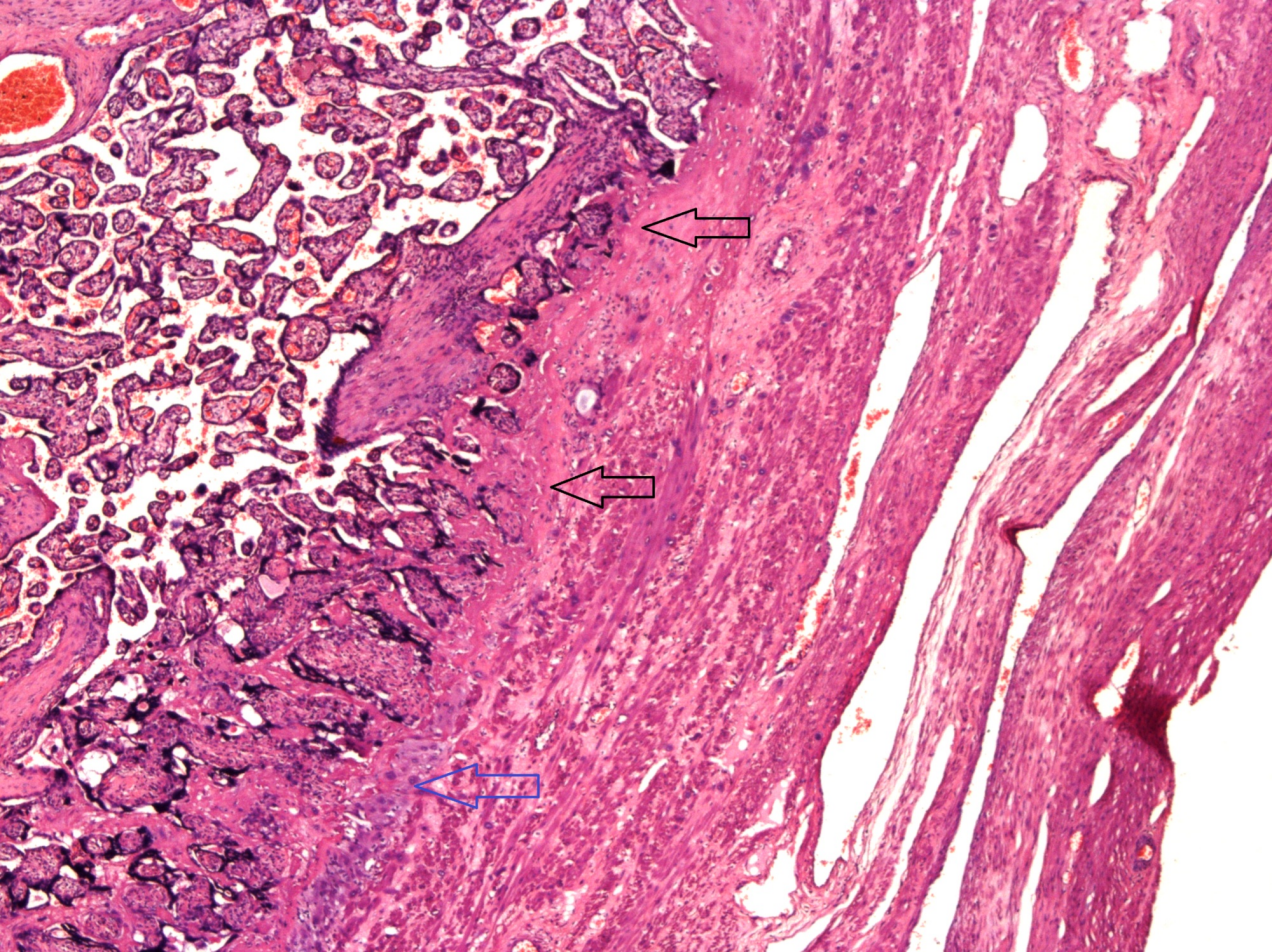
Placenta accreta: lack of decidua between the villi and myometrium. Decidua is shown by the blue arrow, whereas, the black arrows highlight the lack of decidua.

**Figure 3:**
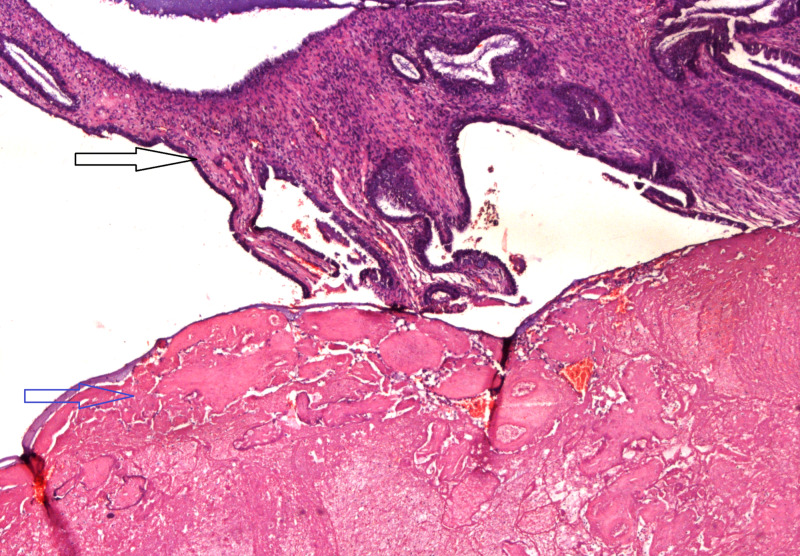
Placenta previa with infarction. Placenta positioned on the endocervix. Black arrow indicates the endocervix, blue arrow shows the infarction of the placental tissue.

**Figure 4:**
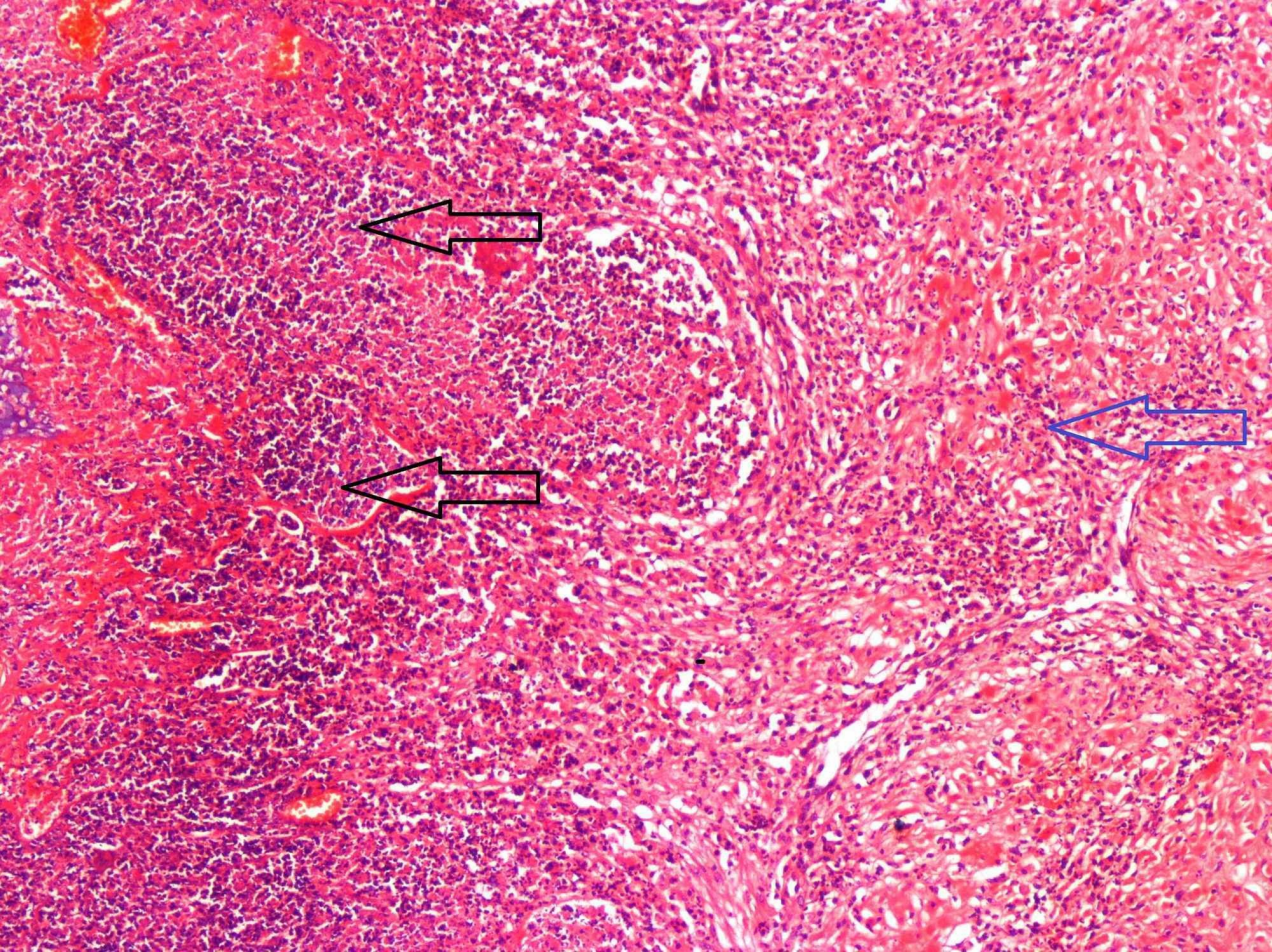
Endomyometritis. Severe inflammation involving the endometrium (black arrows) and myometrium (blue arrow).

**Figure 5:**
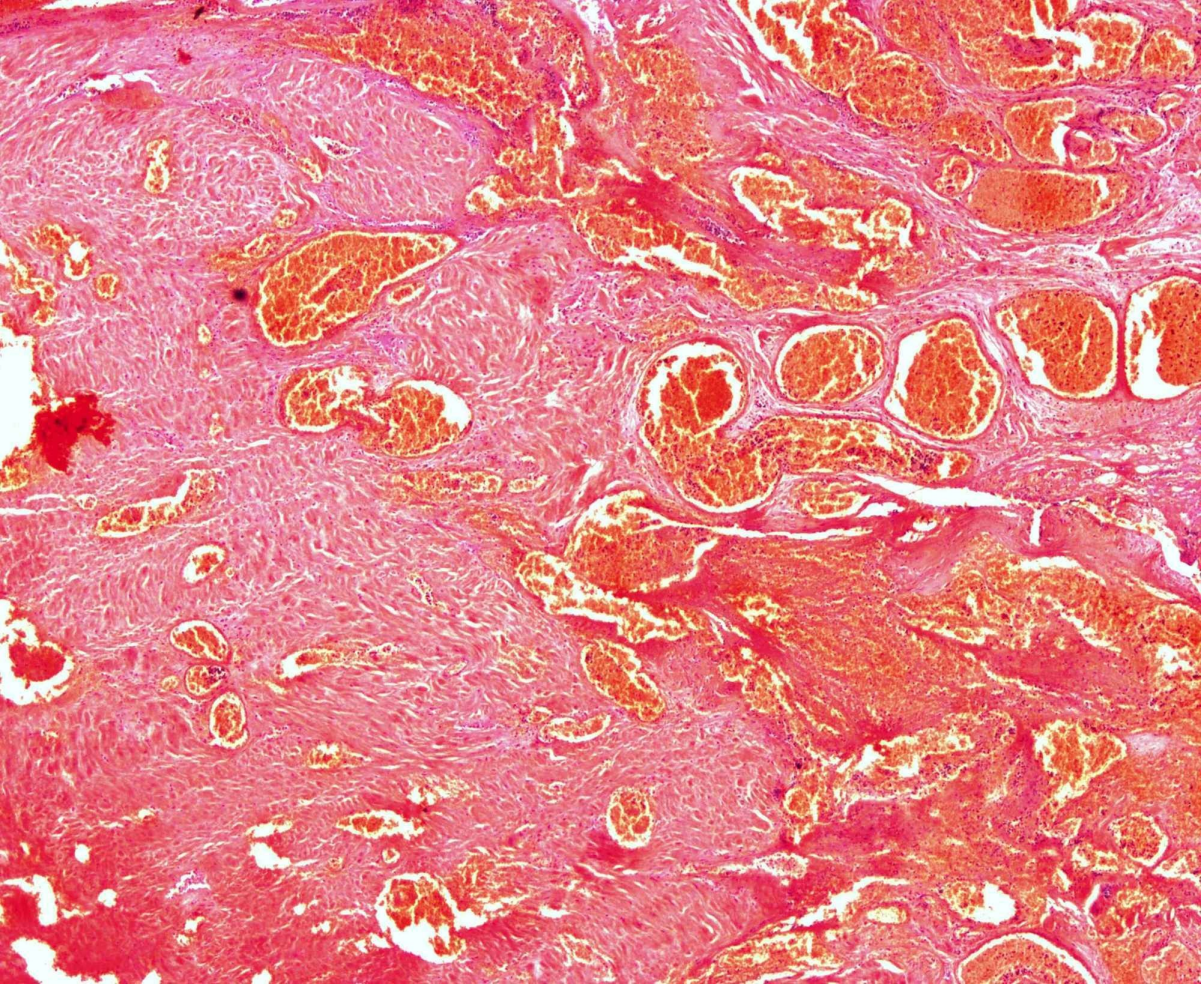
Placental site subinvolution. Subinvoluted, dilated and thrombosed blood vessels in the post-partum placental bed.

**Figure 6:**
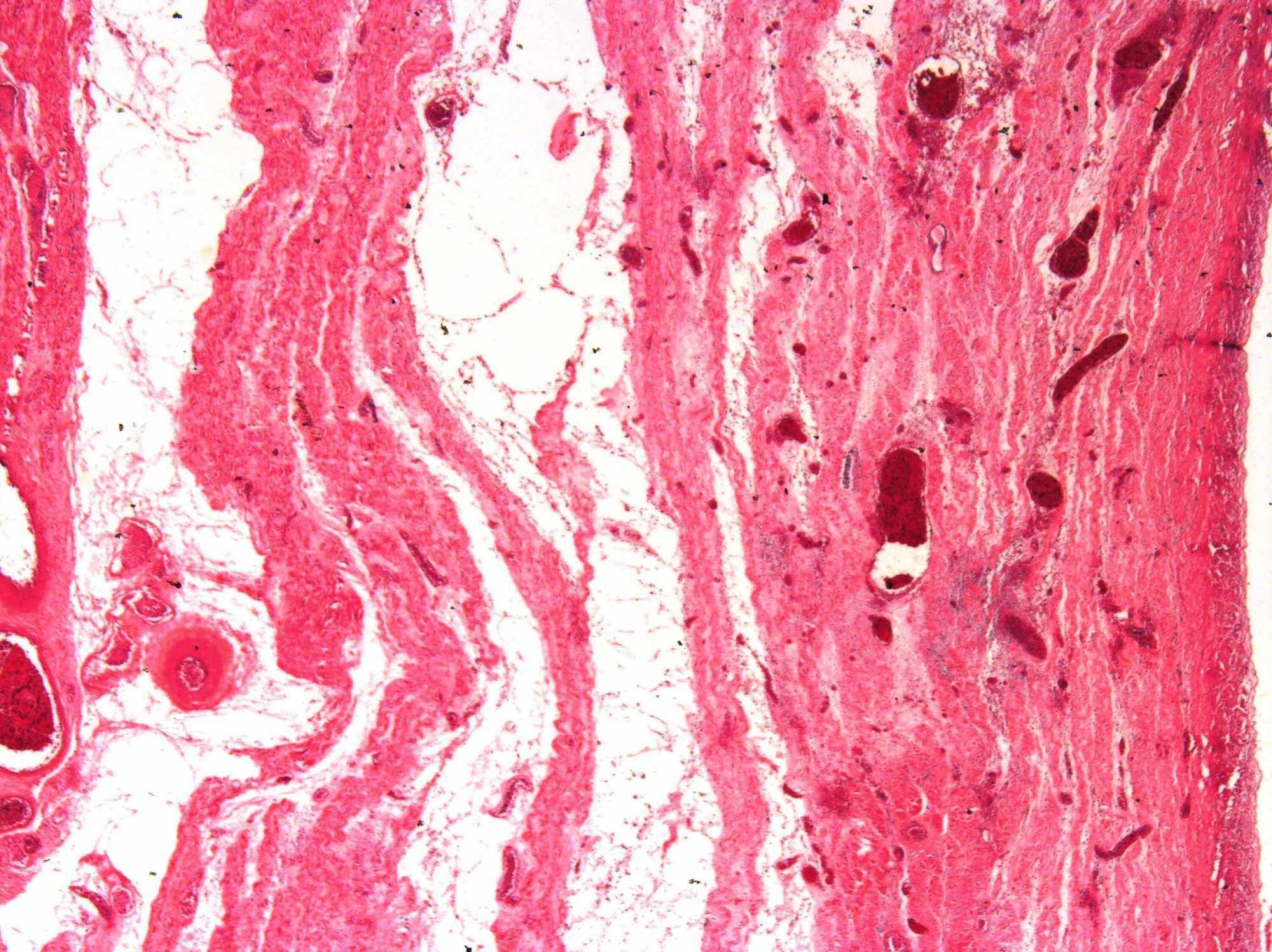
Uterine inertia. Edematous myometrium with dilated and ectatic blood vessels.

Placenta accreta spectrum was the most frequent histopathological finding in the EOH specimen, diagnosed in 61 (63.54%) cases; of the spectrum, placenta accreta was diagnosed in 23 (23.96%) cases, followed by placenta increta in 17 (17.71%) cases and percreta in 10 (10.42%) cases (Figures 1, 2). Eleven cases of placenta accreta spectrum (11.46%) were associated with concomitant placenta previa (Figure 3). Trauma during the delivery was a clinical indication for hysterectomy in eight cases (8.33%) of cervical tear and seven cases (7.29%) of uterus rupture, all of these cases were confirmed on histopathology. In six cases (6.25%) puerperal sepsis was the clinical diagnosis, and histopathology proved these to be endomyometritis (Figure 4). Single case of placental site subinvolution (1.04%) and two cases of uterine inertia (2.08%) were diagnosed (Figures 5, 6).

Twenty placentas were received with the hysterectomies, most of these were morbidly adherent placenta belonging to the accreta spectrum cases. The most common pathology found in these placentas was infarction in eight (40%) cases, followed by intervillous fibrin in six (30%) cases (Table 3) (Figures 1, 3).

**Table 3:** Histopathological findings in placentas received with the obstetric hysterectomies.

Histopathological findings in placentas (n = 20)	Frequency	Percentage
Infarction	8	40.0%
Intervillous fibrin	6	30.0%
Normal	3	15.0%
Inflammation	2	10.0%
Hemorrhage	1	5.0%
Total	20	100.0%

In 11 (11.46%) cases, salpingo-oophorectomy was performed, four (4.16%) cases of bilateral and seven (7.29%) cases of unilateral oophorectomy were received with the hysterectomy specimens. In two cases of unilateral oophorectomy, mature cystic teratoma was diagnosed. Rest of the ovaries had normal histology or functional cysts (corpus luteum or follicular cysts) (Table 4).

**Table 4:** Histopathological findings in ovaries.

Histopathological findings in ovaries (n = 11)	Frequency	Percentage
Functional cysts	7	63.64%
Normal histology	2	18.18%
Teratoma (mature cystic)	2	18.18%
Total	11	100.0%

Eleven fallopian tubes were received, single case of salpingitis was diagnosed, and rest had normal histology.

## Discussion

In the present study the incidence of obstetric hysterectomy was 58.37/10,000 deliveries. The mean age of the patients was 30.59 (±4.62) years. The results of the study established that the placenta accreta spectrum was the chief histopathological diagnosis in 61 (63.54%) cases of EOH. Placenta accreta was the leading entity in the spectrum, followed by increta and percreta. Overlapping features were seen in some cases of placenta accreta spectrum, where placenta accreta and percreta were observed along with placenta previa. Trauma at the time of delivery was also diagnosed in the form of cervical tear in eight (8.33%) and uterine rupture in seven (7.29%) cases. Infection or sepsis was clinically indicated and was confirmed as endomyometritis in six (6.25%) cases.

Variable rates of EOH have been reported world-wide. The rate of 58.37/10,000 deliveries in this study is comparable to 60/10,000 deliveries reported in a previous study from Civil Hospital Karachi, although another study from Pakistan has reported much higher rate of 10.52/1000 deliveries [13,14]. Incidence reported from India is 8.3/10,000, Nigeria 20/10,000, Europe 3.5/10,000, China 6.3/10,000, New Zealand 4/10,000 and from the USA is 7.7/10,000 deliveries [1,8,11,15-17]. Disparity in the rates of obstetric hysterectomies between the countries highlights the differences in the quality of obstetric care. The high rates of EOH reported from Pakistan are mostly from the large tertiary care hospitals. These hospitals receive a substantial number of cases which are referred from the smaller units, private practices and complicated home deliveries. The problem is compounded by illiteracy and poor socioeconomic conditions of the patients [13, 14].

In our study placenta accreta spectrum was the most common pathology. This finding is in contrast to most of the studies from Pakistan which have listed uterine rupture to be the most common indication for hysterectomy. A study from Karachi has reported uterine rupture in 47.1%, atony in 28.8%, and placental causes in 11.6% of cases [7]. Previous study from our institution has reported uterine rupture in 34% and placental causes in 32% of EOH [13]. Study from Abbottabad by Khan et al. has reported uterine rupture in 34.86% of cases, uterine atony in 29.8% and placental accreta in 8.7% of cases [14]. Uterine rupture has also been reported as the leading indication of hysterectomy in a study from Nigeria, Africa, the reason being prolonged obstructed labor [8]. Uterine rupture is associated with multi-parity, augmented labor and previous CS with scar dehiscence and leads to high maternal and fetal mortality [8]. Most studies from India have reported post-partum uterine inertia or atonic hemorrhage as the most common cause of EOH; in contrast, uterine inertia was histologically diagnosed in only two of our cases [1,18]. Uterine inertia results from failure of the uterine muscles to contract during the third stage of delivery and leads to torrential blood loss, and has been associated with sepsis, anemia, previous CS, obstructed labor and multiparty [10]. However, all of these above studies are clinical studies which have reported clinical indications for EOH.

Only few studies have reported histopathological findings in the EOH specimen, these include two studies from India, both of these have reported abnormal placentation to be the leading histopathological finding in 43.7% and 33.4% of EOH specimen, respectively [10,19].

Internationally, placental abnormalities have been reported as the leading cause of postpartum hemorrhage (PPH) and hysterectomy in 53.1% of obstetric hysterectomies in a study from China, 70% from the study from New Zealand and in 44% from Poland [15,16,20]. The most common indication for obstetric hysterectomy in the past was uterine inertia, which has now been replaced by placenta accreta spectrum [21]. The reason for a decrease in the incidence of uterine inertia is the availability of uterotonic drugs and the use of modern techniques such as, arterial embolization and B-Lynch sutures [22]. The increasing world-wide incidence of cesarean section (CS) is now being considered to be responsible for the rising rate of placenta accreta spectrum, formerly known as morbidly adherent placenta [22]. CS scar in the uterus is regarded as the most crucial cause in this regard as human embryo preferentially implants at the scar site at lower uterine segment as it requires low oxygen tension for its growth, leading to accreta and previa; however, further research into the molecular mechanisms is desired [23]. A retrospective study conducted in Italy, spanning over four decades, concluded that the incidence of placenta accreta had increased from 0.12% in 1970s to 0.31% in 2000s at the same time the incidence of CS increased from 17% to 64% [24]. In Pakistan, the proportion of births delivered by C-section has rapidly increased in the past five years, from 14% in 2013 to 22% in 2018 [25]. Although relevant history related to parity and any previous CS was lacking in most of our cases, leading to inability to evaluate risk factors for EOH, but the present study still reports a very high frequency of pathologically confirmed placenta accreta spectrum.

Placenta accreta spectrum can be diagnosed early in pregnancy using ultrasound, which has an overall sensitivity of 90.72% (87.2-93.6%, confidence interval of 95%) and specificity of 96.94% (96.3-97.5%) for its detection and is recommended as the first line investigation. Magnetic resonance imaging may be used for difficult cases and for detection of depth of invasion in placenta percreta [26]. Therefore, management of these cases includes planned early CS at 34-36 weeks of gestation under an expert multi-disciplinary team of obstetrician, surgeon, anesthesiologist, neonatologist and availability of maternal and neonatal intensive care and transfusion facilities [26,27]. Conservative management of placenta accreta spectrum such as leaving the placenta in-situ approach, manual removal of placenta and conservative surgeries are available for women who wish to preserve fertility and are recommended by International Federation of Gynecologists and Obstetricians. Therefore, the prenatal diagnosis of placenta accreta is highly desirable, as it improves patients’ outcome and reduces morbidity and mortality and ultrasound scan is highly accurate in diagnosis in skilled hands [27].

Placentas that were received with the EOH belonged to the placenta accreta spectrum, mostly to placenta percreta cases and showed intervillous fibrin in 30% (Figure 1) of the cases and infarction in 40% (Figure 3), whereas, inflammation in the form of intervillositis and chorioamnionitis was identified in 10%. Placental infarction and fibrin are suggestive of maternal vascular insufficiency, which may lead to adverse fetal outcome such as fetal growth restriction and fetal death [12]. However, fetal outcome was not known in this study and the number of placentas was also limited to form a definite conclusion. Most of the national and international studies reporting on EOH have not reported placental findings. A study showed that women undergoing hysterectomy for uterine atony showed infection and inflammation of placenta when compared to obstetric hysterectomy for other causes [28]. Further research regarding placental changes in EOH and placenta accreta spectrum, correlating with both maternal and fetal outcomes, is recommended.

In this study, oophorectomy was performed in 11 (11.46%) cases, bilateral oophorectomy was performed in four cases (4.16%) and all cases showed normal histology. Out of seven (7.29%) cases of unilateral oophorectomy mature cystic teratoma was diagnosed in two cases. In studies from Pakistan, Khan et al. have reported one case of ovarian malignancy as an indication for EOH [14]. Korejo et al. have reported ovarian conservation in all cases, whereas, Siddiq et al. have not reported oophorectomy with their cases of EOH [7,13]. In most of the Indian studies, adnexa have not been reported [1,10,18,19]. A study from Poland has reported oophorectomy in 15% of cases due to neoplastic condition in the ovaries or hematoma of the peritoneum [20]. Most of patients in the current study are young as indicated by an average age of 30.59 years. Ovaries are an important source of hormones and oophorectomy deprives the young females of important hormonal support, accelerates aging, and increases the risk of cardiovascular diseases, osteoporosis, fractures and psychological conditions as depression, anxiety and substance abuse [29]. It is, therefore, suggested that ovarian conservation should be performed in case of EOH, if there is no clinical or surgical indication for oophorectomy.

The strength of the present study is that it is a histopathology-based study conducted in a large tertiary care hospital. The study confirms the important causes of obstetric hysterectomies and also highlights the histopathological findings in placentas and ovaries. The limitation of the study is that clinical information on the history of patients, maternal and neonatal outcome is lacking and that the duration of the study is small. Prospective multi-centered cohort studies are recommended for confirmation of our findings.

## Conclusions

The study underscores that placenta accreta spectrum is the leading histopathological diagnosis in emergency obstetric hysterectomies. The study emphasizes the need for regular antenatal follow-up and radiological examination of the pregnant women for early detection of placenta accreta spectrum and prevention of obstetric complications, thus reducing maternal and fetal mortality and morbidity. Placental infarction is the most common pathology in the placentas. Ovaries primarily displayed normal histology, suggesting the need for ovarian conservation in cases having no surgical indication for oophorectomy.
